# Effect of silver nanoparticles and REP-PCR typing of *Staphylococcus aureus* isolated from various sources

**DOI:** 10.1038/s41598-024-71781-w

**Published:** 2024-09-23

**Authors:** Eman M. Elghazaly, Helmy A. Torky, Rasha Gomaa Tawfik

**Affiliations:** 1 Microbiology Department, Faculty of Veterinary Medicine, Matrouh University, Matrouh, Egypt; 2 Microbiology Department, Faculty of Veterinary Medicine, Alexandria University, Alexandria, Egypt

**Keywords:** Ag-NPs, REP-PCR, Electron microscopy, Biofilm, COVID-19, Morel's diseased, Microbiology, Molecular biology

## Abstract

This is the primary study at Matrouh Governorate to unveil antibiotic resistance, biofilm formation, silver nanoparticles (Ag-NPs) effect using electron microscopy, and REP-PCR analysis of *Staphylococcus aureus* strains isolated from COVID-19 patients, contaminated food, and Morel’s diseased sheep and goats. A total of 15 *S. aureus* strains were isolated; five from each of the COVID-19 patients, Morel's diseased sheep and goats, and contaminated food. All strains were considered multidrug-resistant (MDR). All strains showed the presence of biofilm. Morphological changes in the cell surface of the bacterium were evidenced, and penetration with the rupture of some bacterial cells. Based on REP-PCR analysis, 4 clusters (C1-C4) with dissimilarity between clusters C1 and C2 8% and between C3 and C4 15%. Cluster I included 3 strains from contaminated food with a similarity of 97%, and Cluster II included 2 strains from contaminated food and 2 from COVID-19-infected patients with a similarity of 96% (confirming the zoonotic nature of this pathogen). Cluster III contained 4 strains isolated from Morel's diseased sheep & goats with a similarity ratio of 99% in comparison the 4th cluster contained 3 strains isolated from COVID-patients and one from Morel's diseased sheep & goats with a similarity ratio of 92%.

## Introduction

*S. aureus* is one of the most important opportunistic pathogens due to its high pathogenicity, high contamination rate for food, rapid transmission, and rapid distribution. This universal microorganism is carried asymptomatically in 20–30% of the human population^1^ and is a risk factor for subsequent infection^2^. The bacterium can cause minor skin lesions to invasive lesions such as pneumonia and endocarditis. The severity of the disease is linked to a battery of virulence factors—more than 70 genes related to its pathogenicity and invasiveness^3^.

Extracellular toxins including *Staphylococcus* enterotoxins (SEs) genes responsible for food poisoning outbreaks^4^. Some of its toxins are linked with an increased death rate among hosts suffering from other diseases. Alpha hemolysin is present in all *S. aureus* strains as the major virulence factor linked with mammary gland necrosis and a higher death rate among infected animals^5^. Contamination of food by methicillin-resistant *S. aureus* (MRSA) strains with a wide range of exotoxins, including enterotoxins confer life-threatening traits on MRSA, thereby making treatment complicated^6^.

The global pandemic illness COVID-19 caused by SARS-CoV-2, also known as COVID-19 by the World Health Organization (WHO)^7^, is characterized by severe acute respiratory syndrome. Today the management for the prevention and control of the disease allows the utilization of vaccines approved by the Food Drug Administration (FDA). Deaths accompanied by respiratory syndrome, endocarditis, and pneumonia draw attention that *S. aureus* may display as a relapse infection by strains responsible for the index case 2 virulent factors and enterotoxins and production of other toxins inhibit the immune response as alarming^8^.

The resistance of *S. aureus* is acquired soon after exposure to treatment with antibiotic^9^ through the acquisition of antibiotics resistance agents, mobile molecular elements such as integrons, and the existence of biofilm. antibiotic resistance poses a threat to food safety and leads to the death of animals^10^.

The mortality of *S. aureus* infections has dramatically increased as a result of the rise of MRSA strains, bringing attention to the requirement for the creation of novel therapeutic and preventive measures such as metallic nanoparticles, exclusively Ag-NPs to counteract multidrug-resistant *S. aureus*^11^. Even though^12^, are convinced that nanoparticles are essential in treatment besides systemic antibiotics to evade colonization of bacteria and possibly septicemia in the clinic; even though its mechanism is not fully known^13^.

Ag-NPs are seen as a good option among nanoparticles with marked antibacterial profiles, relatively inexpensive to produce^14^. Nanotechnology has potential use in human and veterinary medicine^15^. It is not surprising to see nanotechnology being employed to tackle the danger of antibiotic resistance because this technology is increasingly being used in medicine. Nanoparticles can be applied in several ways to cure illnesses^16^.

Repetitive element sequences spread throughout the chromosome of all bacteria and Van Der Zee et al.^17^ suggested REP-PCR genotyping technique based on the presence of homologous *Mycoplasma pneumonia* repeat-like elements in *S. aureus* for tracing the source of infection. Monitoring the prevalence of nosocomial *Staphylococcus* infections is simple and quick. MRSA isolates linked to outbreaks were discovered to share a cluster of identical REP-PCR profiles^17,18^. Manga and Vyletelova^19^ mentioned its higher discriminatory power and reproducibility than RAPD and PFGE.

The main objectives of this research are studying the antibiotics resistance pattern of *S. aureus* isolates, evaluating the effect of silver nanoparticles on pan-drug resistant *S. aureus* strains by electron microscopy and determining the genetic relatedness among *S. aureus* strains isolated from COVID-19 patients, contaminated food and Morel's diseased sheep and goat by REP-PCR at Matrouh Governorate.

## Results

Out of 215 samples (120 contaminated food samples, 65 Morel's diseased sheep, and goats samples, and 30 samples from COVID-19 patients), 15 (7%) *S. aureus* strains were recovered, 5 (16.7%) from COVID-19 patients, 5 (7.7%) from Morel's diseased sheep and goats, and 5 (4.2%) from contaminated food as shown in Table 1. *Staphylococcus* enterotoxin a (sea) was found in four isolates from Morel's diseased sheep and goats, three isolates from the COVID-19 patients had the *Staphylococcus* enterotoxin b (seb) gene, and only one isolate from contaminated food had the seb gene. All strains displayed tolerance to at least three classes of antibiotics. Most strains showed MDR, and only 1(6.67%) of them was Pan-drug resistant (PDR) (showed resistance to six classes of antibiotics) as shown in Tables 2, 3 and all 15 (100%) strains were biofilm producers (6 weak, 4 moderate, and 5 strong) as shown in Table 2, the most pan-drug resistant strain that showed resistance to different six classes of antibiotics also, showed the presence of integron1 (this strain was used for evaluating the effect of silver nanoparticles by electron microscopy).

**Table 1:** The incidence of *Staphylococcus aureus* among different sources of samples.

			Outcome		Total
			Negative	Positive	
Source of sample	COVID 19 human	Count	25	5	30
		% within samples types	83.3%	16.7%	100.0%
	Contaminated food samples	Count	115	5	120
		% within samples types	95.8%	4.2%	100.0%
	Morel’s diseased sheep and goats	Count	60	5	65
		% within samples types	92.3%	7.7%	100.0%
Total		Count	200	15	215
		% within samples types	93.0%	7.0%	100.0%

**Table 2:** Antibiotic resistance pattern and biofilm grade of *Staphylococcus aureus* isolates.

Number of examined isolates	Source	Resistance profile	Resistance of antimicrobial classes	Biofilm grade (OD)
1	Morel's diseased sheep	CF, P, AMX, CL, TE	3 classes	Weak (+ or 1)
2	Morel's diseased sheep	MUP, P, GEN and AMX, TE	3 classes	Weak (+ or 1)
3	Morel's diseased goat	MUP, AMX, P, CF, K, V, TE	4 classes	Moderate (+ + or 2)
4	Morel's diseased sheep	GEN, K, AMX, P, CL, CF, T IMI, T	5 classes	Strong (+ + + or 3)
5	Morel's diseased goat	AMX, CF, V	3 classes	Weak (+ or 1)
6	Positive COVID-19 patient	K, AT, CF, GEN, P, V, AMX	5 classes	Strong (+ + + or 3)
7	Positive COVID-19 patient	AMX, CAZ, CF, MUP, TMP-SMZ	4 classes	Moderate (+ + or 2)
8	Positive COVID-19 patient	AT, MUP, CMP	3 classes	Weak (+ or 1)
9	Positive COVID-19 patient	CEZ, GEN, K, V, P, AMX	4 classes	Moderate (+ + or 2)
10	Positive COVID-19 patient	K, GEN, P, AMX, CL	3 classes	Weak (+ or 1)
11	Contaminated food	CAZ, AT, P, V, CIP, TMP-SMZ,GEN, TE	6 classes	Strong (+ + + or 3)
12	Contaminated food	MUP, CL, P, V, CAZ	5 classes	Strong (+ + + or 3)
13	Contaminated food	K, GEN, AMX, P, V	3 classes	Weak (+ or 1)
14	Contaminated food	TMP-SMZ, AMX, P, V, CAZ	4 Classes	Moderate (+ + or 2)
15	Contaminated food	CAZ, CEZ, CF, AMX, P, V, IMI, GEN, K	5 classes	Strong (+ + + or 3)

**Table 3:** Incidence of pan-drug resistant strain among 15 *S. aureus* isolates of different sources.

Sources of *S. aureus* isolates	Total NO. of *S. aureus* isolates	NO. of pan-drug resistant strains	% of pan-drug resistant strains
COVID 19 human	15	0	0%
Contaminated food samples		1	6.67%
Morel’s diseased sheep and goats		0	0%

% acc. to total NO. of *S. aureus* isolates.

**Fig. 1:**
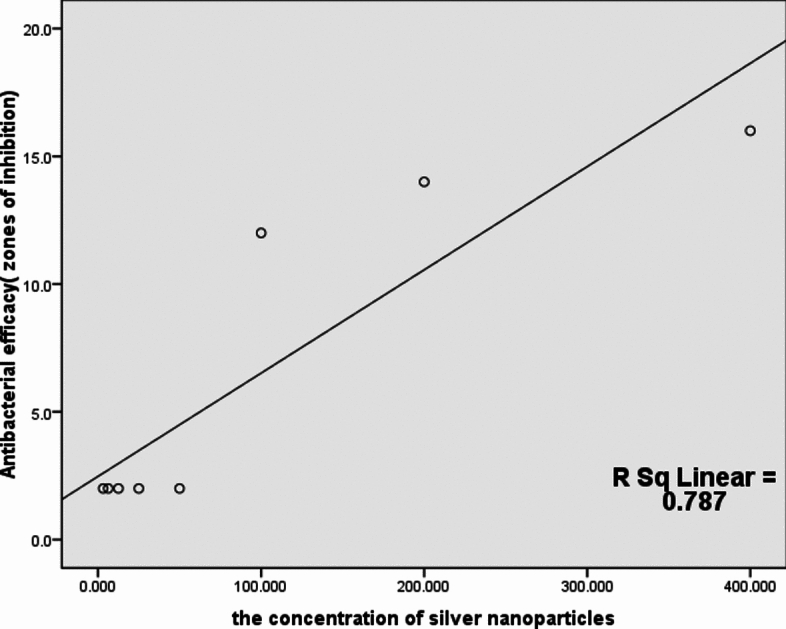
Correlation between silver nanoparticles concentration and antibacterial efficacy.

**Fig. 2:**
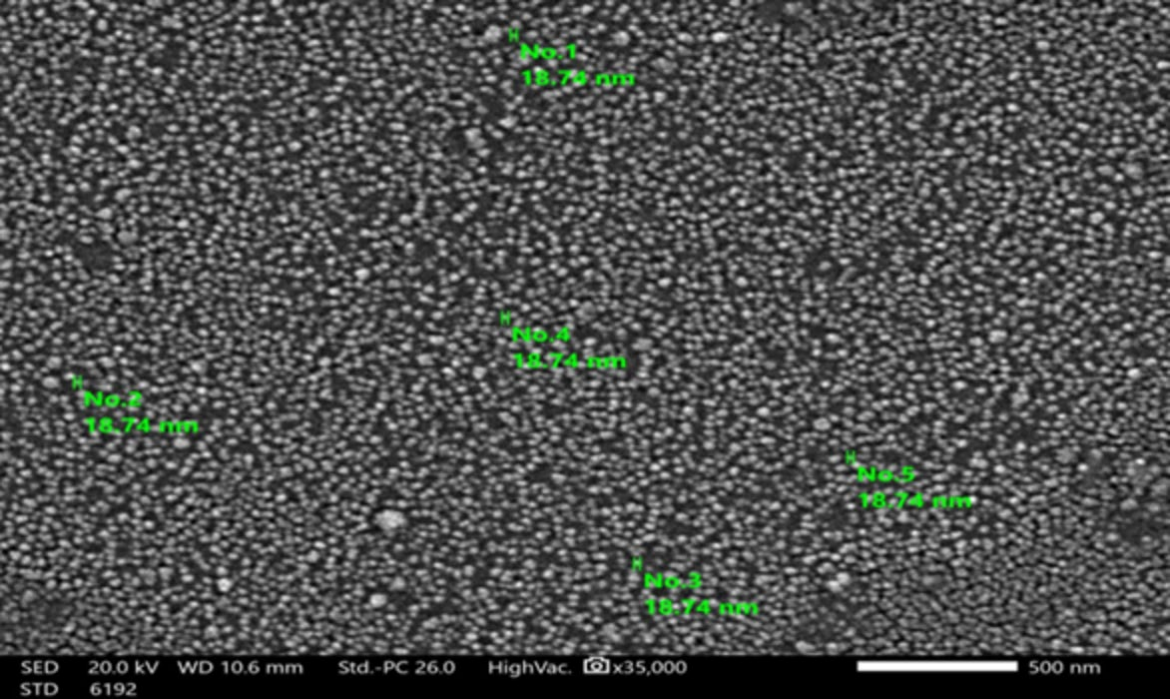
SEM micrograph of spherical Ag-NPs particles and with an 18.7 nm average size.

**Fig. 3:**
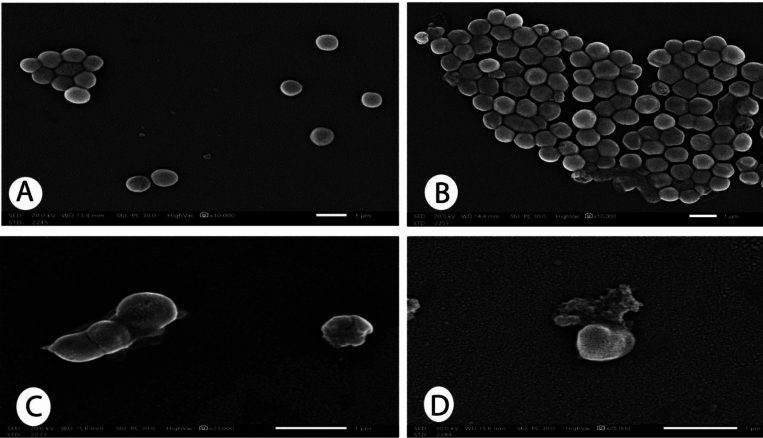
Effect of silver nanoparticles on isolated *S. aureus*. (**A**) The surface of untreated *S. aureus* cells was smooth and retained their coccus/round morphology. (**B**) *S. aureus* cells treated with 100 µg/ml Ag-NPs for 2.5 min appeared to undergo slight lysis. (**C**) *S. aureus* treated with 100 µg/ml Ag-NPs for 10 min appeared to undergo moderate lysis (**D**) *S. aureus* treated with 100 µg/ml Ag-NPs for 25 min appeared to undergo severe lysis, so the contents of the cell were released into the cytoplasm.

**Fig. 4:**
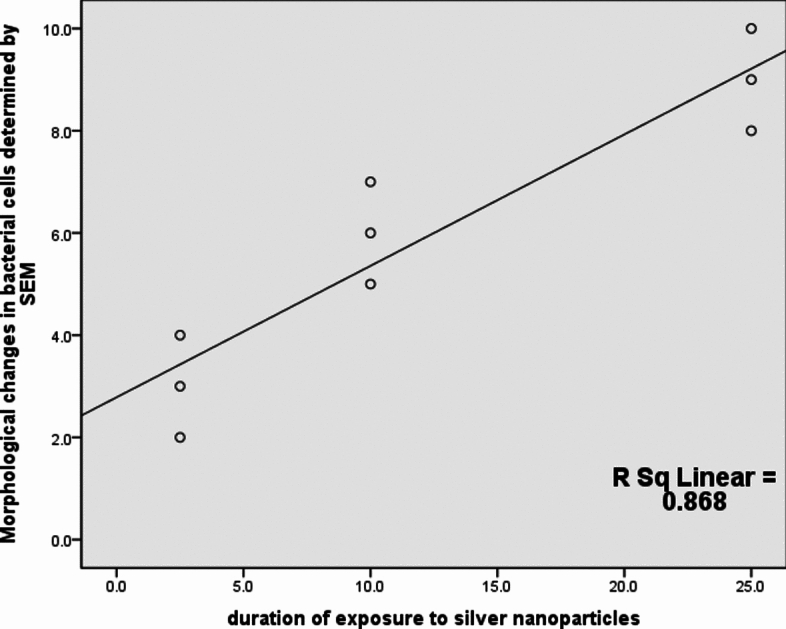
Correlation between duration of exposure to silver nanoparticles and morphological changes in bacterial cells that detected by SEM.

The disc diffusion of Ag-NPs revealed that 100 µg/ml is the minimum bactericidal concentration (MBC) required to inhibit cells of the *S. aureus* strain that exhibited resistance to six classes of antibiotics (pan-drug resistant). The minimum inhibitory concentration (MIC) was 50 µg/ml. The diffusion plate results revealed a strong positive correlation between the concentration of Ag-NPs and antibacterial efficacy, as measured by the zones of inhibition; increasing Ag-NPs concentrations increases the antibacterial efficacy as shown in Table 4 and Fig. 1. The silver nanoparticles (Ag-NPs) used were spherical (Fig. 2). As shown in Fig. 3, the images produced by the presence of Ag-NPs at concentrations of 100 µg/ml and various exposure times of 2.5, 10, and 25 min indicate changes in the integrity of the outer bacterial cell membrane and a strong positive correlation was found between the duration of exposure to silver nanoparticles and the morphological changes in bacterial cells that determined by SEM (Fig. 4) and Table 5, as the effect was more pronounced where the concentration was 100 µg/ml with a 25- min exposure time as shown in Fig. 3D.

**Table 4:** Correlation between silver nanoparticles concentration and antibacterial efficacy.

Kendall's tau_b correlation		Antibacterial efficacy ( zones of inhibition)
The concentration of silver nanoparticles	Correlation coefficient	0.887**
	Sig. (2-tailed)	0.003

**Correlation is significant at the 0.01 level (2-tailed).

**Table 5:** Correlation between duration of exposure to silver nanoparticles and morphological changes in bacterial cells that detected by SEM.

Kendall's tau_b correlation		Morphological changes in bacterial cells determined by SEM
Duration of exposure to silver nanoparticles	Correlation coefficient	0.932**
	Sig. (2-tailed)	0.000

**Correlation is significant at the 0.01 level (2-tailed).

**Fig. 5:**
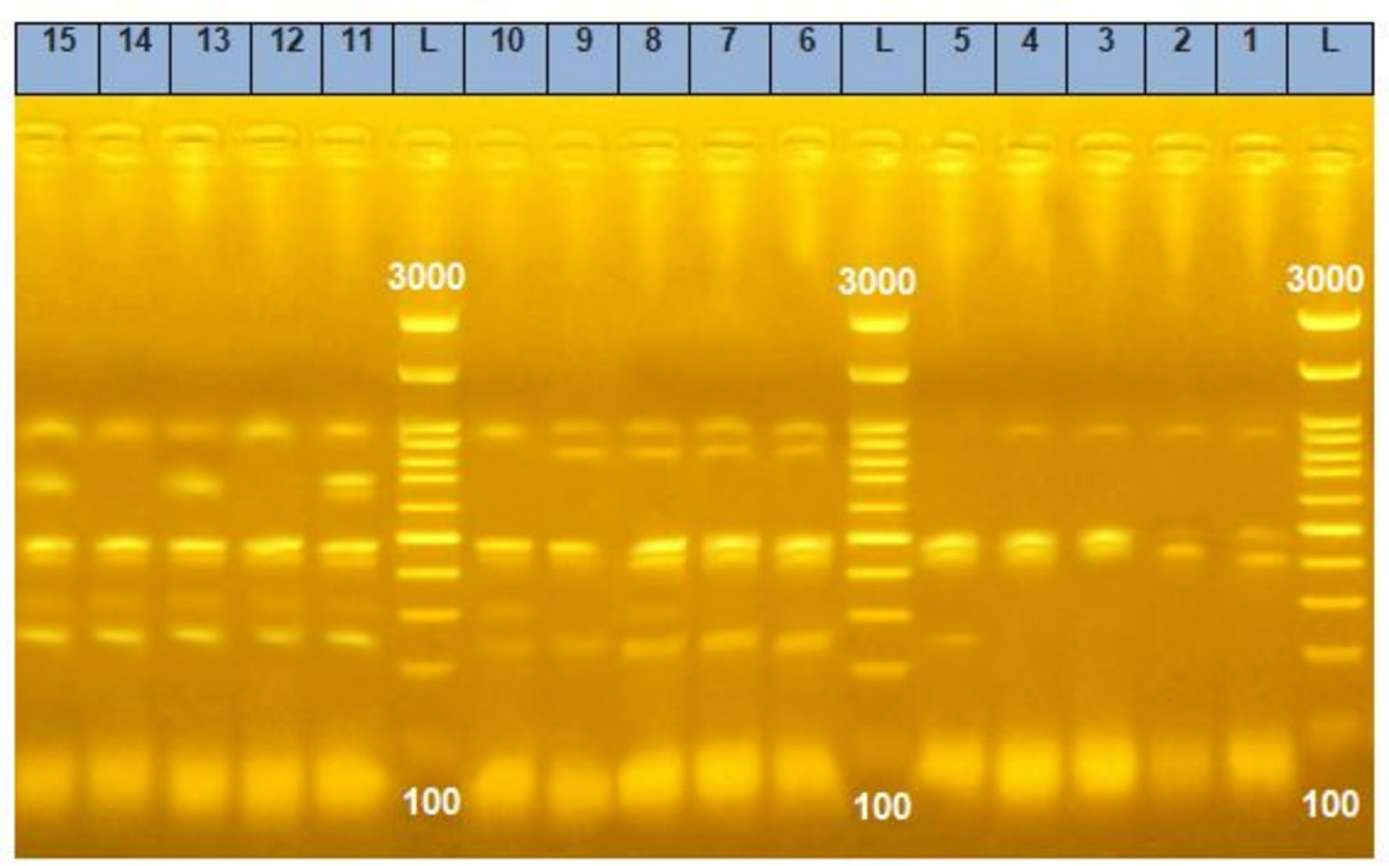
REP-PCR of *S. aureus*. REP-PCR assay of *S. aureus* strains from different sources in 1.5% agarose gel, L: 100 bp molecular marker, lanes (1–5): *S. aureus* isolates from Morel's diseased sheep and goat origin, lanes (6–10): *S. aureus* isolates from positive COVID -19 patient origin, Lanes (11–15):* S. aureus* isolates from contaminated food origin.

**Fig. 6:**
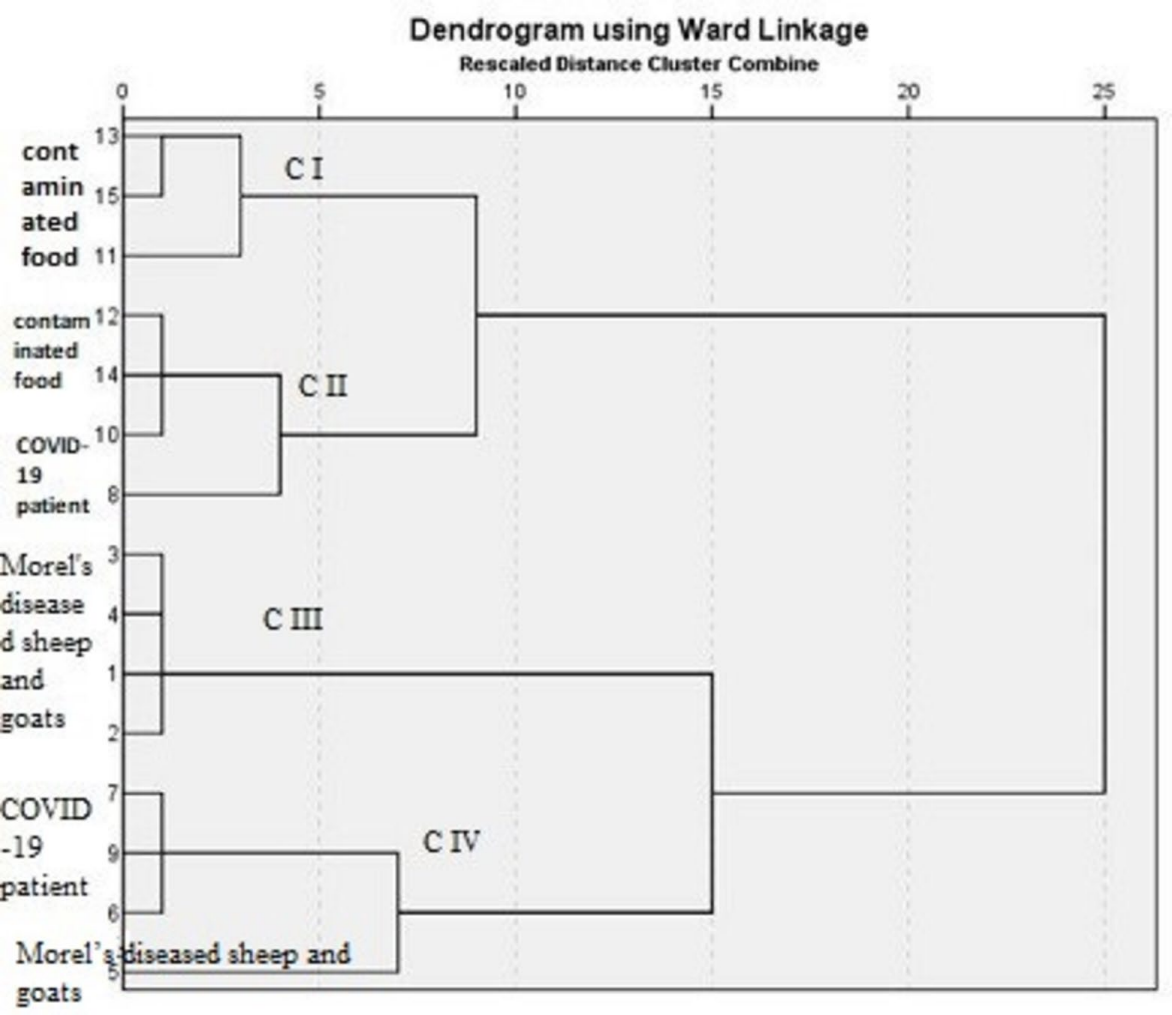
Cropped figure of dendrogram showing the genetic relatedness among 15 *S. aureus* isolates using Ward linkage, (1, 2, 3, 4 and 5): *S. aureus* isolates from Morel's diseased sheep and goat origin, (6, 7, 8, 9 and 10): *S. aureus* isolates from positive COVID-19 patient origin, (11, 12, 13, 14 and 15):* S. aureus* isolates from contaminated food origin.

REP-PCR analysis revealed the presence of 4 clusters and 3 single strains (11, 8, and 5); cluster I included two strains (13 and 15) recovered from contaminated food and a single strain (11) which also, recovered from contaminated food with a similarity ratio about 97% between them, cluster II included 3 strains (12, 14, 10), two of them (12 and 14) recovered from contaminated food and one of them (10) recovered from COVID-19 patients and a single strain (8) that recovered from COVID-19 patients with a similarity ratio about 96%, cluster III which contained 4 strains (1, 2, 3, 4) recovered from Morel's diseased sheep and goats with a similarity ratio about 99% and cluster IV which contained 3 strains recovered from COVID-19 patients (6, 7, 9) and a single strain (5) which recovered from Morel's diseased sheep and goats with a similarity ratio about 92%, the dissimilarity between cluster I (CI) and C II about 8%, but the difference between C III and C IV about 15% as shown in Figs. 5, 6 and Table 6.

**Table 6:** The genetic relatedness among 15 *S. aureus* isolates.

Sources of *S. aureus* isolates	Clustering by ward method				Total	Monte Carlo Sig. (2-sided)	
	Cluster 1	Cluster 2	Cluster 3	Cluster 4			
Morel's diseased sheep and goats	0	0	4	1	5	X2	P
COVID-19 patients	0	2	0	3	5	14.243	0.001
Contaminated food	3	2	0	0	5		
Total	3	4	4	4	15		

## Discussion

*S. aureus*, a symptomatically colonizing normal flora of the skin and nasopharynx of animals and humans, characterized by its pathogenicity for both^20^ causes skin infection, pneumonia, and endocarditis in humans^21^.

In this study, we isolated and identified phenotypically and genotypically 15 *S. aureus* strains out of 215 different samples: five from each of the COVID-19 patients, Morel's diseased sheep and goats, and contaminated food samples.

Enterotoxin not only suppresses the immune system^8^ but also increases the risk of a chronic infection^11^ and is linked to the virulence and pathogenicity of strains^22,23^. Treatment becomes more difficult when any kind of SE acts as a superantigen on polymorphonuclear cells, causing the production of the IL-4 and IL-10 genes and then stimulating Th2 cells^24^. This makes it more difficult to eradicate invasive infections, which can cause serious lung damage in people with cystic fibrosis^25^ and lead to 94,000 invasive diseases and over 18,000 deaths each year in the United States^26^. Every year, 150,000 MRSA infections occur in Europe, and 7000 deaths are associated with these infections^27^**.**

Most isolated strains have one of the SEs. MRSA is the leading bacterium causing food poisoning outbreaks and is regarded as the most prevalent pathogen in human nosocomial infections^28^. 14 (41.2%) of the 34 *S. aureus* isolates were positive for *Staphylococcus* enterotoxin B (SEB)^29^*.*

In this study, all 15 *S. aureus* strains showed resistance to at least three different classes of antibiotics. Researchers determined that 24%, 47%, 91%, 82%, 59%, and 47% of *S. aureus* isolates were resistant to several antibiotics, indicating MDR^29^. All *S. aureus* strains showed different resistance standards to antibiotics^30^, while most of them were MDR that resist penicillins and their derivatives and methicillin, and many of the most frequently recommended beta-lactam antibiotics, comprising amoxicillin and oxacillin, match what is known as MRSA^31^.

All strains (100%) were biofilm producers. Neopane et al.^32^ found that 86.7% of biofilm-producing *S. aureus* exhibited MDR. Moreover, only one isolate showed the presence of integron 1 (the one that showed resistance to six classes of antibiotics). The class 1 integron gene cassette with variable amplicon and the integrase gene (intl1) were found to be present in 42% and 36% of the isolates, respectively^33^. The presence of integron contributes to the transfer of antibiotic-resistance genes among *Staphylococci*^34^.

The condition is exacerbated if contaminated food of animal origin (milk, meat) with *S. aureus* is submitted to patients in poor health^35–37^, which leads to subsequent dissemination of resistance to humans from the food chain, increasing the difficulty of treatment infection in humans and decreasing possible therapeutic uses^20^.

The emergence of resistance to antibiotics that are either not licensed for use in animals or that are listed for use in humans only complicates the issue of antimicrobial resistance in low-source countries like Egypt^38^. Most developed nations have antimicrobial drug resistance (AMR) surveillance and monitoring systems that are routinely updated^7^, as the Danish Integrated Antimicrobial Resistance, Monitoring, and Research Programme in Denmark and the National Antimicrobial Resistance Monitoring Systems (NARMS) in the United States. As a result, these nations have detailed maps of the AMR phenomenon^39^. Alternatively, due to a lack of surveillance networks, laboratory capacity, and proper diagnosis, and consequently, a lack of information, there is no regular surveillance or monitoring system for MDR in developing nations in Africa^40^.

The utilization of metallic nanoparticles that show antimicrobial activities is one of the recent techniques to overcome microbes and multi-drug resistant bacteria^41^. In our investigation, silver nanoparticles (Ag NPs) inhibited the growth of strong biofilm-forming and pan-drug-resistant *S. aureus*. In disc diffusion tests, the increase in the concentration of Ag-NPs increases the antibacterial efficacy; this relation was noticed by Armanullah et al.^42^ in Gram–negative bacteria. There is a direct relation between the duration of exposure to silver nanoparticles and its effect on the microorganisms, as found by Li et al.^43^ who reported that the exposure of *S. aureus* cells to 50 g/ml Ag-NPs for 6 h caused the DNA to become condensed and lose its capacity for replication, while its exposure to 50 g/ml Ag-NPs for 12 h caused the cell wall to break down, allowing the contents of the cells to leak out into the environment. Eventually, the cell wall disintegrated. The enzymatic activity of respiratory chain dehydrogenase may lso be decreased by Ag-NPs due to the low diffusibility of engineered nanoparticles (ENP). The efficacy of disc diffusion susceptibility assays for evaluating their antibacterial activity is in doubt, but Kourmouli et al.^44^ proved that their penetration through the cultured media and Ag-NPs' unique size-dependent properties are not responsible for their antibacterial diffusion behavior, which is instead ascribed to the ions they release.

The mechanisms of Ag NPs' action are blurred and unclear. The exact mechanisms are not completely understood. According to Duran et al.^13^ and Abdelrehiem et al.^45^, Ag-NPs may interact with the bacterial cell wall, produce reactive oxygen species (ROS), interact with DNA, and release Ag^+^ ions^41^. According to Qing et al.^46^, dissolving Ag^+^ ions produced during the oxidation and dilution of Ag-NPs in an aqueous solution and their subsequent reaction with cell membrane proteins are what cause the morphological changes seen in bacteria exposed to Ag-NPs^47^, reported that nanoparticles may be able to interact with the cell wall of bacteria by changing lipopolysaccharide and making pores that alter the cell membrane while Vazquez-Munoz et al.^48^ attributed its action to the direct influence of silver on membrane stability, an anchor to the bacterial cell wall, allow Ag-NPs to penetrate the intracellular environment that achieves to enter the cell cytoplasm and release Ag^+^ that can interact with various biomolecules ended by bacterial death.

By using electron imQaging, we were able to demonstrate that Ag-NPs accumulated on the cell wall of *S. aureus*, penetrated the interior, and interacted with biomolecules, possibly causing the cell wall to rupture and/or bacterial death. In the twentieth century, people thought that silver was comparatively nanotoxic to mammalian cells, aside from the fact that it could lead to argyria. However, research has shown that silver-based compounds can be significantly toxic to human and animal cells at the nanoscale. These problems must be addressed before people hurry to indulge in the nanosilver boom^49^.

Finally, based on the results of REP-PCR as a recommended tool for partial fingerprinting analysis of 15 *S. aureus* strains, it revealed the presence of 4 clusters, gave detailed information to enable comparative analysis, confirmed the zoonotic nature of this important pathogen, and shared a common source, contamination, and/or infection. El-Gedawy et al.^50^ reported that the four Rep-PCR primers generated roughly 55 fragments, of which 29 (52.5%) are considered to be polymorphic bands among *S. aureus* isolates and 26 (47.5%) are considered to be monomorphic bands. Holmes and Zadoks^34^ provide evidence that humans are the primary natural carriers of *S. aureus*, which can contaminate food and put customers at risk for health problems. Shepheard et al.^51^ reported that animal *S. aureus* strains are distinct from those infecting humans, and Manga and Vyletelova^19^, concluded that REP-PCR is good compared to RAPD and PFGE, the pulsed-field gel electrophoresis screening of 42 *S. aureus* strains yielded six clusters. Abdelrahman^52^ subtyped 12 strains of *S. *aureus (10 from ruminants and 2 from poultry) at 75% genetic similarity into 6 ERIC-types (A1:A3, B1:B3). Holmes and Zadoks^34^ attributed the diversity to random *nuc*leotide mutations and horizontal gene transfer.

## Conclusion

In conclusion, the powerful biofilm-forming and antibiotic-resistant *S. aureus* strain development is inhibited by Ag-NPs. There is a direct relationship between the concentration and the antibacterial efficacy, as well as, between the duration of exposure to silver nanoparticles and their effect on the microorganisms. REP-PCR is one of the most effective methods to determine the genetic relatedness between different strains.

## Material and methods

### Sampling

#### Sample size

Two hundred and fifteen samples (120 contaminated food samples, 65 Morel's diseased sheep and goats samples, and 30 samples from COVID-19 patients) were collected from Matrouh Governorate for the isolation of *S. aureus*. By using the Epi Info sample size calculator, the odds ratio of contaminated food samples was 0.217 and the infection rate was 15%, so the minimum sample size was 106 contaminated food samples, the odds ratio of Morel's diseased sheep, and goats was 0.417 and the infection rate was 54%, so the minimum sample size was 56 Morel's diseased sheep and goats, the odds ratio of COVID-19 human was 0.200 and the infection rate was 22%, so the minimum sample size was 27 COVID-19 human cases^53–55^, so the minimum number required for doing our study was 189 samples. The odd ratios of different samples were illustrated in Table 7.

**Table 7:** Exhibition of the Logistic regression to predict each sample type's risk factors (odds ratio).

	B	S.E	Wald	df	Sig	Exp (B) (odds ratio)	95.0% C.I. for EXP(B)		Classification Table	Hosmer and Lemeshow Test	Nagelkerke R Square
							Lower	Upper			
Contaminated food samples	− 1.526	0.670	5.190	1	0.023	0.217	0.058	0.808	93%	1.000	0.057
Morel’s diseased sheep and goats	− 0.875	0.676	1.678	1	0.195	0.417	0.111	1.567			
Constant	− 1.609-	0.490	10.793	1	0.001	0.200					

References category COVID 19 human.

#### Isolation of *S*. *aureus*

For the isolation of *S. aureus*, nasopharyngeal swab samples were taken from COVID-19 patients, Morel's diseased sheep, and goats^1^. Briefly, from clinical cases, cotton-tipped swabs moistened with sterile saline, rolling over the lesion surface five times, focusing on the area where there was evidence of pus or an inflamed area, and contaminated food samples were processed as recorded by Artursson et al., Wang et al.^56,57^. Using Mannitol salt agar (Oxoid), Baired- parker agar (Hi- media, Mumbai), and oxacillin resistance screening agar base plate (ORSAB) (Oxoid, UK) (it gives characteristic dense blue colonies)^58^.

#### Identification of *S. aureus*

Identification of *S. aureus* was carried out by microscopic examination, colonial characteristics, and biochemical identification: catalase-positive, coagulase-positive, and oxidase-negative^58^, besides species-specific *nuc* gene PCR. Also, PCR investigation to its enterotoxin gene presence (Table 8). All molecular characterization was performed at the Animal Health Research Institute, Dokki, Giza, Egypt.

**Table 8:** Target genes, Primer sequences, amplicon sizes, cycling conditions of *S. aureus* enterotoxin genes and integron 1.

Target gene	Primers sequences	Amplified segment (bp)	Primary denaturation	Amplification (35 cycles)			Final extension	Reference
				Secondary denaturation	Annealing	Extension		
*nuc*	GTGCTGGCATATGTATGGCAATTG	660 bp	94˚C 2 min	98˚C 10 s	58˚C 30 s	68˚C 1 min	68˚C 7 min	^59^
	CTGAATCAGCGTTGTCTTCGCTCCAA							
*Sea*	GGTTATCAATGTGCGGGTGG	102	94˚C 5 min	94˚C 30 s	57˚C 30 s	72˚C 30 s	72˚C 7 min	^60^
	CGGCACTTTTTTCTCTTCGG							
*Seb*	GTATGGTGGTGTAACTGAGC	164			57˚C 30 s	72˚C 30 s	72˚C 7 min	
	CCAAATAGTGACGAGTTAGG							
*Sec*	AGATGAAGTAGTTGATGTGTATGG	451			57˚C 40 s	72˚C 45 s	72˚C 10 min	
	CACACTTTTAGAATCAACCG							
*Sed*	CCAATAATAGGAGAAAATAAAAG	278			57˚C 30 s	72˚C 30 s	72˚C 7 min	
	ATTGGTATTTTTTTTCGTTC							
*See*	AGGTTTTTTCACAGGTCATCC	209			57˚C 30 s	72˚C 30 s	72˚C 7 min	
	CTTTTTTTTCTTCGGTCAATC							
*Integron (hep 35 and hep 36 primers)*	TGCGGGTYAARGATBTKGATTT	491			55˚C 40 s	72˚C 45 s	72˚C 10 min	^61^
	CARCACATGCGTRTARAT							
*Class 1 Integron cassettes*	GGCATCCAAGCAGCAAG	Variable			55˚C 40 s	72˚C 45 s	72˚C 10 min	^62^
	AAGCAGACTTGACCTGA							

### Antimicrobial susceptibility testing

The disc diffusion technique^63^ using a bacterial suspension with turbidity standards of 0.5 McFarland and Muller Hinton agar plates (OXOID) using 16 antibiotics of different classes: CEZ (cefazolin), CF (Cefoxitin), CAZ (Ceftazidime), AMX (Amoxicillin), AT (Azithromycin), TMP-SMZ (Sulpha/Trimethoprim),CMP (Chlorampheniol), IMI (Imipenem), CIP (Ciprofloxacin), GEN (Gentamycin), CL (Clindamycin), K (Kanamycin), MUP (Mupirocin), P (Penicillin), V (Vancomycin) and TE (Tetracycline), and the diameter of inhibition zone was estimated as described by Christensen et al.^64^.

### Biofilm formation

The Micro titer plate method for recognizing biofilm formation was performed following the scheme mentioned by Christensen et al.^64^. By using a micro-ELISA auto- reader at a wavelength of 570 nm, the optical density of the adherent stained biofilm was measured, and the results are determined according to the following equations, as shown in Table 9.

**Table 9:** The equations used to determine the biofilm grade of isolates.

NO	Results	Equation
1	Non biofilm producer (0)	$\text{OD}\le \text{ODc}$
2	Weak biofilm producer(+ or 1)	ODc > OD $\le$ 2 × ODc
3	Moderate biofilm producer(+ + or 2)	$2\times \text{ODc}<\text{OD}\le 4\times \text{ODc}$
4	Strong biofilm producer (+ + + or 3)	$3\times \text{ODc}<\text{OD}\le 4\times \text{ODc}$

### Detection of integron

The selected pan-drug-resistant strain was selected for detection of integron using hep 35 and hep 36 genes, which encoded a conserved region of the integrase gene^61^ and then for detection of integron 1 cassette^62^ using PCR-specific primers as shown in Table 8. It was carried out at the Animal Health Research Institute, Dokki, Giza, Egypt.

### Preparation and characterization of silver nanoparticles^65^

#### Preparation of silver nanoparticles

Using starch as a means of decreasing and stabilizing the obtained Ag-NPs, high throughput production was used to prepare the Ag-NPs solution^66^. In summary, 2 g of raw rice starch (WINLAB laboratory chemicals Co., U.K.) was progressively dissolved with stirring in an alkaline solution pH 11 (0.3 g sodium hydroxide; Sigma-Aldrich, Germany). The mixture was continuously stirred until the starch was completely dissolved. Concurrently, 100 ml of deionized water were used to dissolve 0.5 g of silver nitrate [99.99%; Sigma-Aldrich, Germany]. At 60 °C, the silver nitrate solution was gradually added to the starch solution while being stirred. The color progressively changed from a murky white to a transparent yellow hue, which is indicative of Ag-NP production. The final concentration of Ag-NPs solution was 400 µg/ml.

#### Characterization of silver nanoparticles

Initially, ultraviolet visible spectroscopy (UV–Vis; TG 80; Germany) was used to measure the absorbance of Ag-NPs. The findings indicate that Ag-NPs have a robust absorption peak at 408 nm, but starch molecules do not exhibit any discernible band that could be linked to surface plasmon excitation. Transmission electron microscopy (TEM; JEOLJEM-1230; Japan) was used to examine the particle form and distribution of starch-mediated Ag-NPs. The resulting particles verified the high capacity of starch to reduce silver ions and stabilize the generated Ag-NPs, with a small spherical size of approximately 40 nm and a well-distributed size. After centrifuging the high throughput Ag-NPs solution for 60 min at 4480 × g, Ag-NPs was obtained as powder. An ambient Siemens D500 X-ray diffractometer (30 mA and 40 kV) with a copper tube was used to examine the elemental analysis of the powdered Ag-NPs using X-ray diffraction (XRD). Ag-NPs demonstrated a highly crystalline face-centered cubic architecture, as corroborated by the strong peaks seen in the XRD at 2θ = 37.88°, 44.27°, 64.43°, and 77.35°. The Ag-NPs (220), (200), (311), and (111) planes are the indexes for the diffraction peaks.

### Determination of *S. aureus* susceptibility/resistance to Ag-NPs

After preparing 0.5 McFarland’s standard from *S. aureus* in Brain heart infusion broth (BHI), A Muller Hinton agar (MH) plate was swabbed with the bacteria suspension, and the plate was then incubated for 30 min. Different concentrations of Ag-NPs diluted in de-ionized water were added to wells in the agar with adequate spacing between wells, and then plates were incubated for 20 h at 37 °C. According to CLSI, the lowest concentration of Ag-NPs that kills 100% of the initial bacterial population (showing no colony on MH agar after 20 h of incubation at 37 °C) is known as the minimum bactericidal concentration (MBC). After detecting the minimum bactericidal concentration of Ag-NPs, the tested bacteria, separately, were inoculated onto brain heart infusion broth and then centrifuged to collect a higher number of pellets. The collected pellets were adjusted to be incubated with Ag-NPs colloidal solution concentration to match the minimum bactericidal concentration of Ag-NPs for 2.5, 10, and 25 min, respectively, to be examined by SEM for morphological changes determination^67,68^.

#### Bacterial growth inhibition test:^69,70^

One colony from the pan drug-resistant strain was inoculated in brain heart infusion broth (BHI) (Sigma Aldrich) and kept for 24–48 h in a shaking incubator (144 rpm). BHI broth was used to dilute the bacterial culture to adjust the count to 10^6^ CFU/ ml. An equal volume of each concentration of nanoparticles diluted solution (400, 200, 100, 50, 25, 12.5, 6.25, 3.12 µg/ml) was added to the same volume of a bacterial strain to obtain a 5 × 10^5^ CFU/ ml total volume. Following overnight incubation in a shaking incubator under the same conditions described above, 100 µl of each sample was streaked in a static incubator to track the bacterial growth curve. The experiment was carried out in triplicate.

#### Determination of the minimum inhibitory concentration (MIC)^71^

Double-fold serial dilution of silver nanoparticles was added to a culture holding 10^6^ CFU/ ml and incubated as before. On Muller Hinton agar plates, 100 µl of each was then smeared. The smallest double-fold serial solution with little to no bacterial growth is the minimal inhibitory concentration (MIC).

### Scanning electron microscope

All the electron microscopy investigation was carried out at the Faculty of Science, Alexandria University. The morphological measurement of nanoparticles was conducted using SEM (Vega Tescan, USA) that measures the size of nanoparticles^72^.

### Ultrastructure observations

By using SEM (scanning electron microscope) at the Faculty of Science, Alexandria University, the particle size, shape of silver nanoparticles and the morphological changes in treated bacterial cells with nanoparticles were determined.

### REP- PCR

Table 10 showed the primer pairs for REP-PCR amplification and conditions. After electrophoresis in a submerged agarose gel (1.5%), the size of the amplified fragments was recognized^73^. Computer software was used to analyze the data. The REP-PCR and the analysis of the data was carried out at the Animal Health Research Institute, Dokki, Giza, Egypt.

**Table 10:** Target genes, primer sequences, amplicon sizes, and cycling conditions of REP-PCR.

Target gene	Primers sequences	Amplified segment (bp)	Primary denaturation	Amplification (35 cycles)			Final extension	Reference
Staph REP				Secondary denaturation	Annealing	Extension		
REP2-I-ICGICTTATCIGGCCTAC		Variable	94˚C 5 min	94˚C 1 min	46 ˚C 1 min	72˚C 2 min	72˚C 12 min	^74^

### Statistical analysis

Data were collected in a spreadsheet Excel, SPSS version 22 was used. Monte Carlo Sig. (2-sided) and Kendall's tau_b correlation were applied to predict the association between independent variables and outcomes, between qualitative variables. Logistic regression was applied to predict the risk factors (odds ratio) of independent variables and outcomes. Dendrogram Ward linkage was used to rescale distance cluster combine.

## Data Availability

Data is provided within the manuscript.
